# Prevalence and associated factors of internet addiction among adolescents during the COVID-19 pandemic in Kota Bharu, Kelantan, Malaysia: A cross-sectional study

**DOI:** 10.51866/oa.469

**Published:** 2024-10-19

**Authors:** Lena Nanditha Sangaran, Azidah Abdul Kadir, Lili Husniati Yaacob

**Affiliations:** 1 MD, M.Med, PhD, Department of Family Medicine, School of Medical Sciences, Health Campus, Universiti Sains Malaysia, Kubang Kerian, Kelantan, Malaysia. Email: azidahkb@usm.my; 2 MD, MMed, Department of Family Medicine, School of Medical Sciences, Health Campus, Universiti Sains Malaysia, Kubang Kerian, Kelantan, Malaysia.; 3 MBBS, M.Med, Department of Family Medicine, School of Medical Sciences, Health Campus, Universiti Sains Malaysia, Kubang Kerian, Kelantan, Malaysia.

**Keywords:** Adolescent, COVID-19, Internet addiction

## Abstract

**Introduction:**

The study aimed to determine the prevalence and associated factors of internet addiction (IA) among early adolescents during the COVID-19 pandemic in Kota Bharu, Kelantan, Malaysia.

**Methods:**

A cross-sectional online survey was conducted from February to April 2021 among adolescents in Kota Bharu, Kelantan. Participants completed the Malay Version of the Internet Addiction Test, the Depression, Anxiety and Stress Scale-21 and a sociodemographic information form. A multiple logistic regression analysis was performed to determine the associations between variables.

**Results:**

A total of 535 adolescents participated in this study. Among them, 65.9% were girls. The prevalence of IA among the participants was 48.6%. Male sex [adjusted odds ratio (AOR)=2.15, 95% confidence interval (CI)=1.30—3.57], internet usage at home (AOR=6.17, 95% CI=1.94—19.58), internet usage to watch/download music (AOR=2.50, 95% CI= 1.52-4.12), internet usage to engage in social networking (AOR=2.29, 95% CI=1.19—4.40), inadequate parental/guardian bonding (AOR=2.19, 95% CI=1.31-3.65), depression (AOR=2.03, 95% CI=1.07—3.85), anxiety (AOR=2.79, 95% CI=1.64—4.76) and stress (AOR=4.67, 95% CI=2.00—10.91) were significantly associated with IA.

**Conclusion:**

IA was prevalent among Malaysian adolescents during the COVID-19 pandemic. Sex, internet usage profile and psychological factors were significant predictors of IA.

## Introduction

The COVID-19 outbreak was declared a worldwide pandemic emergency by the World Health Organization (WHO), and several countries across the globe developed and implemented strategies such as movement restriction orders and even total lockdowns to mitigate the epidemic curve.^1^ This critically affected the educational system, as schools were temporarily shut down so students could stay home and follow social distancing measures.^2^

The COVID-19 pandemic has instigated a digital revolution in many sectors and caused an upsurge in internet usage trends in adults and adolescents. A recent survey conducted in Indonesia noted that adolescents comprised the largest proportion of internet users.^3^ The introduction of online learning during the movement restriction order in the country yielded a rise in internet use for information, education and communication among adolescents. The internet was also used as a mode of entertainment and withdrawal from stress and anxiety and as a means to alleviate a depressed mood due to school closure, which significantly disrupted the typical adolescent lifestyle.^4^ While reasonable internet use is beneficial, excessive uncontrolled utilisation can lead to internet addiction (IA).^5^ IA is defined as individuals’ inability to control their use of the internet, which consequently causes psychological, social, school-related and work-related difficulties.^5^ Among Asian countries, China and Taiwan identified IA as a worsening public health issue during the COVID-19 pandemic.^6,7^

Adolescents may experience mental health issues such as loneliness, boredom, depression, anxiety and stress due to a lack of emotional support and spiritual guidance from parents, teachers and other peers as they are confined to their homes.^7^ A drastic increment in the prevalence of depression from 36.6% to 57% and anxiety from 19% to 36.7% during the early period of the COVID-19 outbreak was seen in a study conducted in China.^8^ An increase in mental health problems during the pandemic may consequently exacerbate IA and vice versa.

A potential risk factor for IA in adolescents is male sex.^9^ Other evidence shows that girls are more prone to IA than boys, as they exhibit problematic smartphone and Facebook usage.^8^ Furthermore, specific internet activities are potentially addictive; individuals become attached not to the medium itself but to the actual behaviours they engage in online, such as social networking, online gaming and online pornography.^9^ Parents’ involvement and parenting type are also important predictors of IA. Previous studies have shown that overprotective, restrictive parenting styles and disorganised family structures are associated with an increased incidence of IA.^10^ Stress, depression and anxiety are also correlated with IA. Excessive internet usage and dependency can result in various psychosocial and health issues, leading to a loss of control over time spent online,^10^ an impairment in academic performance,^11^ a lack of physical activity^10^ and the development of musculoskeletal disorders.^11^ Adolescents are a vulnerable age group that can be easily exploited into addictive behaviours without recognising the possible adverse consequences.^12^ However, to our knowledge, there is a paucity of data on IA and its relationship with psychological disorders and substance abuse among adolescents. Thus, this study aimed to determine the prevalence and associated factors of IA among adolescents in Malaysia. The findings will be used to spur and guide a national policy on mental health and addiction, particularly IA during and after the COVID-19 outbreak.

## Methods

### Study design

A cross-sectional study was conducted from February to April 2021 among adolescents in lower secondary government schools in Kota Bharu, Kelantan, on the east coast of Peninsular Malaysia. Students aged 13-14 years attending day schools were included. Students attending boarding schools or programmes for those with mental disabilities and students with known psychiatric illnesses were excluded. The sample size was calculated to meet all study objectives, with the largest sample size selected using a single-proportion formula to determine the prevalence of IA. Based on a prevalence of IA of 10%, a precision of 0.05 and a non-response rate of 50%, the final sample size was calculated as 560.

### Data collection

Seven secondary schools were randomly chosen via two-stage stratified cluster sampling. The first and second sampling stages involved the selection of schools and classes, respectively. Permission to conduct the study was obtained from the Ministry of Education Malaysia and the principals of the chosen schools.

An online questionnaire survey was conducted via Google Forms. The study was initially advertised via the online teaching group of chosen classes on Telegram and WhatsApp. Interested students were invited to join the survey through the Google Form link attached to the advertisement. The online questionnaire contained information about the study, including its summary, its potential benefits and risks and confidentiality of collected data. Parents and students were required to complete an online informed consent form, confirming their understanding of the purpose, eligibility criteria, risks and benefits of the study. Thereafter, students were invited to complete a series of questionnaires on their own. Those who had no parental consent or those who did not provide assent were directed to end the survey without answering any further questions and were not included in the study.

### Research instruments

***Sociodemographic and internet usage profile questionnaire***

A sociodemographic and internet usage profile questionnaire was prepared in the Malay language. It assessed students’ age and sex, parents’ marital status, mother’s/father’s employment status, household income and internet usage information such as tools for access, place of access and motive for use.

***Malaysian Global School-Based Student Health Survey***

A questionnaire from the Malaysian Global School-Based Student Health Survey was used in this study.^13,14^ The original questionnaire was developed by the WHO and the Centers for Disease Control and Prevention in collaboration with the United Nations Children’s Fund, the United Nations Educational, Scientific and Cultural Organization and the Joint United Nations Programme on HIV and AIDS in 2001. Since then, the questionnaire has been used in many countries globally to obtain data on important behaviours and protective factors among adolescents.^13^

The Malay version of the questionnaire was developed by the Ministry of Health Malaysia and the WHO in 2012. It was used in the Malaysia Adolescent Health Survey in 2017.^13^ The questionnaire consists of five items: tobacco use, alcohol consumption, drug use, physical activity and parental relationship. Each item has multiple-choice answers that range from a ‘no’ response to various frequencies of behaviours, including the age at which each behaviour started.

***Malay Version of the Internet Addiction Test (MVIAT)***

The validated MVIAT was used in this study. The Internet Addiction Test was developed in 1998 by Dr Kimberly Young.^5^ Guan et al. validated the Internet Addiction Test in the Malay language in 2015 and named it the MVIAT.^15^ This test views IA as an impulse control disorder and refers to the internet as encompassing all types of internet activity. The MVIAT consists of 20 items that evaluate the level of IA. Specifically, these items measure behavioural, personal, occupational and social functioning problems associated with compulsive use of the internet. A 5-point Likert scale *(1 = rare, 5=always)* is used to rate each item. The lowest possible score is 20, while the highest possible score is 100, with higher scores indicating greater levels of IA. The optimal cutoff score for distinguishing between students with IA and non-internet addiction (NIA) was 43. The test showed good internal consistency (Cronbach’s *a* coefficient=0.91, P<0.001), parallel reliability (intra-class correlation coefficient=0.88, P<0.001) and concurrent validity with the Compulsive Internet Use Scale (Pearson’s correlation coefficient=0.84, P<0.001).^15^

***Depression, Anxiety and Stress Scale-21 (DASS-21)***

This study utilised the Malay version of the DASS-21, which was previously validated and used among a similar age group in the Adolescent Health Survey in 2017.^16^ The scale has three subscales, each of which contains seven items: depression, anxiety and stress. Each item is scored on a 4-point Likert scale ranging from 0 *(did not apply to me at all)* to 3 *(applied to me very much).* A depression score of 14 or more, an anxiety score of 10 or more and a stress score of 19 or more are considered to indicate depression, anxiety and stress, respectively.^16^ The Malay validation demonstrated acceptable Cronbach’s a coefficients of 0.84, 0.74 and 0.79 for depression, anxiety and stress, respectively.^16^

### Statistical analysis

Data were entered and analysed using the Statistical Package for the Social Sciences version 25.0. (IBM SPSS Statistics for Windows, Version 25.0. IBM Corp., Armonk, NY.) Data checking and cleaning were performed before analysis. The quantitative variables were presented using means and standard deviations (SDs) and the categorical variables as frequencies and percentages. Multiple logistic regression was used to determine the associated factors of IA. Simple logistic regression was employed to analyse the categorical variables. All variables with a P-value less than 0.25 were included in the multiple logistic regression analysis. Backward and forward stepwise procedures were performed for all variables significantly related to IA. The final model was adjusted for confounders, specifically age and sex, to determine the independent effect on IA. The associations were expressed as odds ratios with their corresponding 95% confidence intervals (CIs). P-values less than 0.05 were considered statistically significant. The goodness of fit of the model was analysed using the Hosmer-Lemeshow test, a classification table and receiver-operating characteristic curves. All tests were two-sided.

## Results

A total of 634 students responded to the survey. However, 99 students were excluded: Five lacked participant assent; 37 lacked parental consent; and 57 did not meet the age criteria. Therefore, the total number of participants analysed was 535. Table 1 shows the characteristics of the participants and their distribution relative to NIA and IA. Of the 535 participants, 182 (34%) were boys, while 353 (65.9%) were girls. The mean age of the participants was 13.5 (SD=0.5) years, with the age range being 13-14 years. The mean total household income was RM 3233.9 (SD=3740.6), ranging from RM 150 to RM 50,000. Most internet users were students from households in the B40 income group (n=381, 71.2%). The prevalence of IA was 46.8% (n=260).

**Table 1:** Comparison of the sociodemographic characteristics, internet use profile and psychological factors between the users with and without internet addiction.

Variable	**Without internet addiction**		**With internet addiction**	
	n 260	%	n 275	%
**Sododem ographic characteristics**				
**Sex**				
Male	73	26.5	109	41.9
Female	202	73.5	151	58.1
**Age (year)**				
13	118	42.9	111	42.7
14	157	57.1	149	57.3
**Internet use profile**				
**Place of access**				
Home	250	90.9	252	96.9
School	9	3.3	29	11.2
Friends or another persons home	26	9.5	47	18.1
Free Wi-Fi anywhere	24	8.7	57	21.9
Commercial internet access facility	17	6.2	51	19.6
**Tools for access**				
Smartphone	247	89.8	252	96.9
Computer/laptop/notebook	62	22.5	85	32.7
Tablet/iPad	15	5.5	42	16.2
**Motive for use**				
Social networking	200	72.7	221	85.0
Email or instant messaging	33	12.0	67	25.8
Obtain information	106	38.5	104	40.0
Study	214	77.8	158	60.8
Watch/down load music/online radio	76	27.6	133	51.2
Watch/down load video/online TV	81	29.5	125	48.1
Online gaming	119	43.3	157	60.4
Online shopping	56	20.4	74	28.5
**Familial factors**				
**Parents’ marital status**				
Married and living together	227	82.5	185	71.2
Married but living apart due to work	9	3.3	16	6.2
Divorced/separated	18	6.5	33	12.7
Widower (mother/father has died)	21	7.6	26	10.0
**Household income per month (MYR)**				
B40 (<RM 3860)	205	74.5	176	67.7
M40 (RM 3860-8319)	53	19.3	67	25.8
T20 (>RM 8319)	17	6.2	17	6.5
**Parental relationships**				
**Parental/guardian supervision**				
Yes	104	37.8	59	22.7
No	171	62.2	201	77.3
**Parental/guardian connectedness**				
Yes	148	53.8	76	29.2
No	127	46.2	184	70.8
**Parenral/guardian bonding**				
Yes	219	79.6	133	51.2
No	56	20.4	127	48.8
**Parental/guardian respect for privacy**				
Yes	60	21.8	39	15.0
No	215	78.2	221	85.0
**Psychologicalfactors**				
**Depression**				
Not depressed	241	87.6	143	55.0
Depressed	34	12.4	117	45.0
**Anxiety**				
Not anxious	218	79.3	113	43.5
Anxious	57	20.7	147	56.5
**Stress**				
Not stressed	265	96.4	171	65.8
Stressed	10	3.6	89	34.2
**Risky behaviours**				
**Smoking**				
Yes	1	0.4	15	5.8
No	274	99.6	245	94.2
**Alcohol use**				
Yes	1	0.4	6	2.3
No	274	99.6	254	98.7
**Illicit drug use**				
Yes	3	1.1	1	0.4
No	272	99.6	259	99.6
**Physical activity**				
No physical activity	117	42.5	128	49.2
Moderate physical activity	98	35.6	95	36.5
High physical activity	60	21.8	37	14.2

Table 2 shows the associated factors of IA based on the simple logistic regression analysis. Male sex was associated with a higher risk of IA. Other factors such as tools for access, place of access, motive for use, inadequate parental supervision, bonding and respect for privacy were also significantly associated with IA. The participants experiencing depression, anxiety and stress and exhibiting risky behaviours such as smoking and physical inactivity showed an increased risk of IA.

**Table 2:** Associated factors of internet addiction among the lower secondary government school students based on the simple logistic regression analysis.

Variable	Crude odds ratio	95% Confidence interval	Wald statistic	P-value
***Sociodemographic characteristics***				
**Sex**				
Male	2.00	1.39-2.87	13.90	**<0.001**
Female	Ref.			
**Age (year)**				
13	Ref.			
14	1.01	0.72-1.42	0.003	0.960
***Internet use profile***				
**Place of access**				
Home	3.15	1.39-7.12	7.61	**0.006**
School	3.71	1.72-8.00	0.39	**0.001**
Friend’s or another person’s home	2.11	1.27-3.53	8.18	**0.004**
Free Wi-Fi anywhere	2.94	1.76-4.90	17.04	**<0.001**
Commercial internet access facility	3.70	2.08-6.60	19.68	**<0.001**
**Tools for access**				
Smartphone	3.57	1.60-7.99	9.60	**0.002**
Computer/laptop/notebook	1.67	1.14-2.45	6.85	**0.009**
Tablet/iPad	3.34	1.80-6.19	14.70	**<0.001**
**Motive for use**				
Social networking	2.13	1.38-3.27	11.72	**0.001**
Email or instant messaging	2.55	1.61-4.02	16.01	**<0.001**
Obtain information	1.06	0.75-1.50	0.12	0.731
Study	0.44	0.30-0.64	17.97	**<0.001**
Watch/download music/online radio	2.74	1.92-3.93	30.31	**<0.001**
Watch/download video/online TV	2.22	1.55-3.16	19.28	**<0.001**
Online gaming	2.00	1.42-2.82	15.51	**<0.001**
Online shopping	1.56	1.05-2.32	4.73	**0.03**
**Familial factors**				
**Parents’ marital status**				
Married and living together	Ref.			
Married but living apart due to work	2.18	0.94-5.05	3.32	0.069
Divorced/separated	2.25	1.23-4.13	6.87	**0.009**
Widower (mother/father has died)	1.52	0.83-2.79	1.83	0.177
**Household income per month (MYR)**			
B40 (<RM 3860)	Ref.			
M40 (RM 3860-8319)	1.47	0.97-2.23	3.38	0.066
T20 (>RM 8319)	1.17	0.58-2.35	0.18	0.670
**Parental relationships**				
**Parental/guardian supervision**				
Yes	Ref.			
No	2.07	1.42-3.03	14.20	**<0.001**
**Parentai/guardian connectedness**				
Yes	Ref.			
No	2.82	1.97-4.03	32.38	**<0.001**
**Parental/guardian bonding**				
Yes	Ref.			
No	3.73	2.55-5.47	45.91	**<0.001**
**Parental/guardian respect for privacy**				
Yes	Ref.			
No	1.58	1.01-2.47	4.08	**0.043**
**Psychologicalfactors**				
**Depression**				
Not depressed	Ref.			
Depressed	5.80	3.76-8.96	62.93	**<0.001**
**Anxiery**				
Not anxious	Ref.			
Anxious	4.98	3.40-7.28	68.14	**<0.001**
**Stress**				
Not stressed	Ref.			
Stressed	13.79	6.98-27.26	56.98	**<0.001**
**Physical activity**				
No physical activity	1.77	1.10-2.87	5.47	**0.019**
Moderate physical activity	1.57	0.96-2.59	3.18	0.075
High physical activity	Ref.			

The results of the multiple logistic regression analysis are shown in Table 3. The analysis revealed that 10 factors were significantly associated with IA. The male participants had a twofold higher risk for IA than the female participants [adjusted odds ratio (AOR)=2.15, 95% CI=1.30—3.57]. The participants who had internet access at home demonstrated a sixfold higher risk for IA than those who had internet access at school (AOR=6.17, 95% CI=1.94—19.58). Conversely, the participants who used the internet to watch/ download music (A0R=2.50, 95% CI=1.52—4.12) and engage in social networking (AOR=2.29, 95% CI=1.19—4.40) had more than a twofold higher risk for IA than those who used it to obtain information.

**Table 3:** Associated factors of internet addiction among the lower secondary government school students based on the multiple logistic regression analysis.

Variable	Adjusted odds ratio	95% Confidence interval	Wald statistic	P-value^a^
**Sex**				
Male	2.15	1.30-3.57	8.80	**0.003**
Female	Ref.			
***Internet use profile***				
**Place of access**				
Home	6.17	1.94-19.58	9.53	**0.002**
School	3.10	1.15-8.33	5.01	**0.025**
Friend’s or another person’s home	-	-	-	-
Free Wi-Fi anywhere	-	-	-	-
Commercial internet access facility	1.94	0.89-4.23	2.77	0.096
**Motive for use**				
Social networking	2.29	1.19-4.40	6.14	**0.013**
Email or instant messaging	1.74	0.94-3.22	3.06	0.080
Obtain information	-	-	-	-
Study	0.20	0.11-0.34	32.40	**<0.001**
Watch/download music/online radio	2.50	1.52-4.12	12.89	**<0.001**
Watch/download video/online TV	1.72	1.04-2.86	4.40	**0.036**
***Familial factors***				
**Parental/guardian bonding**				
Yes	Ref.			
No	2.19	1.32-3.65	9.11	**0.003**
***Psychological factors***				
**Depression**				
Not depressed	Ref.			
Depressed	2.03	1.07-3.85	4.66	**0.031**
**Anxiety**				
Not anxious	Ref.			
Anxious	2.79	1.64-4.76	14.28	**<0.001**
**Anxiety**				
Not stressed	Ref.			
Stressed	4.67	2.00-10.91	12.68	**<0.001**

a Hosmer–Lemeshow test: P<0.05; classification table: 79.6%; The Area Under the ROC Curve (AUC)=550.278

The participants who perceived inadequate parental/guardian bonding showed a more than twofold higher risk for IA than those who perceived adequate parental/guardian bonding (AOR=2.19, 95% CI= 1.31-3.65). The psychological factors also predicted IA. The participants who were depressed had a twofold higher risk for IA than those who were not depressed (AOR=2.03, 95% CI= 1.07-3.85), while the participants who were anxious had an almost threefold higher risk for IA than those who were not anxious (AOR=2.79, 95% CI= 1.64—4.76). The participants who were stressed demonstrated a fivefold higher risk for IA than those who were not stressed (AOR=4.67, 95% CI=2.00-10.91). Conversely, the participants who accessed the internet to study had an 80% lower risk for IA than those who used the internet to obtain information (AOR=0.20, 95% CI=0.11-0.34).

## Discussion

The present study showed that the prevalence of IA among adolescents in Malaysia was 48.6% during the COVID-19 pandemic, which is relatively high compared to 29.0% reported in a survey conducted in 2017 among Malaysian adolescents.^13^ The prevalence is also higher than that observed in recent studies during the pandemic in Taiwan (24.4%)^6^ , Vietnam ( 20.9%)^17^ and Indonesia (19.3%).^18^ This might be due to the increased internet reliability for daily living activities during the COVID-19 pandemic following school closures and movement control orders (MCOs). MCOs were a series of national quarantine measures that were commonly referred to globally as ‘lockdowns’. Nonetheless, internet use at home yielded a higher risk for IA than internet access at schools and commercial facilities.^19^ This might be directly related to the MCO implementation, in which adolescents spent most of their time at home.

The prevalence of IA in this study is comparable to that in another study conducted among 500 secondary school children in Sabah and Johor (45%). The latter study was conducted in 2022, when there was a transition from a pandemic to an endemic state, while our study was conducted in 2021, when the school system had a total lockdown.^20^ The findings are also comparable to those of a systematic review of 11 articles on the prevalence of IA during the pandemic (62%). However, this review did not specify studies among adolescents.^21^ Conversely, a study conducted among adolescents during the pandemic in China revealed that the prevalence of IA was 50.6%,^22^ which is comparable to our data. Taken together, these findings provide evidence that links the pandemic with internet-related addictive behaviours such as IA. There were several reasons for the upsurge of this prevalence during the pandemic. Several studies have reported a relationship between psychological disorders such as depression and anxiety and IA.^22,23^ Specifically, the pandemic increased the prevalence of psychological disorders among adolescents due to various reasons such as disruptions to the educational system, social interruptions, limited social activity and sharing of limited house space with other family members.^23^ There was a vicious cycle in which depressive symptoms and IA disorders upsurged each other during the pandemic.^22^

Some demographic variables were discovered to be associated with a high prevalence of IA. In this study, the male participants were found to be at a higher risk for IA than the female participants. This may be explained by differences in internet use preferences between the sexes. For example, male individuals are more likely to engage in online entertainment and leisure than female individuals.^9^ Conversely, our study showed that the participants who used the internet to watch/download music/online radio, watch/ download music/online TV and engage in social networking had a higher risk for IA. These findings are consistent with those of a study conducted during the pandemic in Indonesia.^18^ In many studies, sex has been investigated as a variable associated with IA. However, despite the mounting research on this issue, a consistent conclusion could not be established. For example, Shek and Yu concluded that male adolescents were at a higher risk of developing IA than female adolescents.^24^ However, another research showed no significant sex difference in IA owing to the current era of easy internet accessibility from anywhere via mobile devices.^25^

In this study, the prevalence of smoking was substantially low at 3%, with only one smoker in the NIA group. This finding is similar to that in a study conducted among Malaysian adolescents before the COVID-19 pandemic.^13^ However, this finding needs to be interpreted with caution, as smoking is a sensitive issue, and there is a possibility that the data were underreported. Adolescents are particularly vulnerable to different addictive behaviours influenced by the internet during this transitional age period.^26^ The National Health and Morbidity Survey 2017 reported that high-risk behaviours, such as smoking, alcohol consumption and illicit drug use, were prevalent among school-age youth.^13^ Since the prevalence of high-risk behaviours was low and could therefore not be analysed, we excluded them from the analysis of associated factors. Further studies are needed to assess these factors in the future.

This study showed a significant risk of IA among the participants who were depressed, anxious and stressed and who perceived inadequate parental bonding. A similar finding was discovered among adolescents in China during the outbreak.^7^ Pandemic situations and school closures negatively impact students, as they lose their emotional and spiritual guidance from teachers and peers while confined to their homes. The easy accessibility and entertaining medium of the internet provide a common way for adolescents to escape reality and manage their emotions and stress. Extended school closures disrupt routines, increase boredom and limit opportunities for innovative engagement in various academic and extracurricular activities. Consequently, some adolescents develop adverse effects due to their inability to play outside, meet friends or participate in school activities.^27,28^

The association of depression, anxiety and stress with IA in this study concurs with the findings of a study conducted in China among 7958 Chinese adolescents before and during the COVID-19 pandemic.^22^ A bidirectional relationship between IA and depressive symptoms and an increasing prevalence of IA over the two pandemic waves were reported.^22^ There are a few explanations for these observations.

Adolescents with depression often use the internet to access supportive social networks and experience a sense of control, all while avoiding their emotional difficulties and escaping from reality. To relieve their internal struggles, they turn to the internet for entertaining activities, calming social networks and anonymous venting.^22^

The association between IA and parental bonding has been reported in a few studies.^29,30^ This finding implies that parents are markedly important in preventing IA among adolescents. Improving parent-adolescent bonding and parental mediation (co-viewing and talking) is crucial in preventing IA.^30^ In March 2020, the Malaysian government enforced an MCO to manage COVID-19 infections. This led to social isolation among adolescents, who spent more time at home with their parents. The results indicate that parents play an important role during such times and should use this opportunity to strengthen parent-adolescent bonding to curb psychological problems. When emotional support from peers and teachers cannot be met due to home confinement, parental support becomes detrimental.^9^ This situation can adversely affect the mental health of adolescents during a pandemic.

This study has some limitations. It was conducted in a single region on the east coast of Peninsular Malaysia with a predominance of Malays, which may not fully represent the country’s multicultural and ethnic diversity. There is also a possibility of pre-COVID memory bias, as the data were self-reported. Additionally, given the cross-sectional nature of the study, the causal influence of various factors such as sex, home internet use for music and social networking, parental bonding, smoking and psychological distress (depression, anxiety and stress) on IA could not be unequivocally determined.

Despite its limitations, the study has important implications for research and intervention, especially during the pandemic. IA has become prevalent among Malaysian adolescents during the COVID-19 outbreak. Sex, internet use profile and psychological factors are identified as significant predictors of IA. However, the extent to which these factors - being male, using the internet at home, using the internet for music and social networking, experiencing inadequate parental/guardian bonding, smoking and facing depression, anxiety and stress - contribute to IA remains unclear for developing effective prevention and intervention programmes in response to COVID-19 and beyond. Nonetheless, these findings provide insights into the risk factors associated with IA, which can help shape targeted prevention and intervention strategies that address its multidimensional nature. These strategies may include parental involvement, education on healthy internet use, mental health support and behavioural interventions to mitigate the negative impacts of excessive internet use and promote wellbeing, especially among adolescents.

This study addresses a gap in the current literature by identifying the prevalence and associated factors of IA during the COVID-19 pandemic among lower secondary school students in Kota Bharu, Kelantan. Understanding the prevalence and associated factors of IA among adolescents during this major period is a preliminary step towards developing effective educational and preventive strategies for adolescents. Further studies should explore the impact of awareness among adolescents, parents and teachers on reducing or preventing IA. This information is expected to assist teachers, schools and the education ministry in providing and regulating effective online learning and mitigating the possible impact of IA among secondary school students.

## Data sharing statement

The datasets generated and/or analysed during the current study are not publicly available, as they are part of a larger study. However, they can be obtained from the corresponding author upon reasonable request.

**How does this paper make a difference in general practice?**

- Internet addiction among early adolescents during the COVID-19 pandemic is concerning.
- Sex, internet use profile and psychological factors significantly influence internet addiction.
- Further studies on the impact of awareness among adolescents, parents and teachers on internet addiction are needed.

## References

[1] Musinguzi G, Asamoah BO (2020). The science of social distancing and total lock down: does it work? Whom does it benefit?. Electron J Gen Med..

[2] Azar AS, Tan NHI (2020). The application of ICT Techs (mobile-assisted language learning, gamification, and virtual reality) in teaching English for secondary school students in Malaysia during COVID-19 pandemic.. Univers J Educ Res..

[3] Duan L, Shao X, Wang Y (2020). An investigation of mental health status of children and adolescents in China during the outbreak of COVID-19.. J Affect Disord..

[4] Chen X, Qi H, Liu R (2021). Depression, anxiety and associated factors among Chinese adolescents during the COVID-19 outbreak: a comparison of two cross-sectional studies.. Transl Psychiatry..

[5] Lebni JY, Toghroli R, Abbas J (2020). A study of internet addiction and its effects on mental health: a study based on Iranian university students.. J Educ Health Promot..

[6] Awaluddin SM, Ibrahim Wong N, Rodzlan Hasani WS (2019). Methodology and representativeness of the Adolescent Health Survey 2017 in Malaysia.. Asia Pac J Public Health..

[7] Rakhmawati W, Kosasih CE, Widiasih R, Suryani S, Arifin H (2021). Internet addiction among male adolescents in Indonesia: a qualitative study.. Am J Men’s Health..

[8] Chao M, Chen X, Liu T, Yang H, Hall BJ (2020). Psychological distress and state boredom during the COVID-19 outbreak in China: the role of meaning in life and media use.. Eur J Psychotraumatol..

[9] Young K (2016). Internet Addiction Test (IAT)..

[10] Lin MP (2020). Prevalence of internet addiction during the COVID-19 outbreak and its risk factors among junior high school students in Taiwan.. Int J Environ Res Public Health..

[11] Kuss DJ, Griffiths MD (2017). Social networking sites and addiction: ten lessons learned.. Int J Environ Res Public Health..

[12] Lam LT (2020). The roles of parent-and-child mental health and parental internet addiction in adolescent internet addiction: does a parent-and-child gender match matter?. Front Public Health..

[13] Gomez P, Rial A, Brana T, Golpe S, Varela J (2017). Screening of problematic internet use among Spanish adolescents: prevalence and related variables.. Cyberpsychol Behav Soc Netw..

[14] Ying Ying C, Awaluddin SM, Kuang Kuay L (2021). Association of internet addiction with adolescents’ lifestyle: a national school-based survey.. Int J Environ Res Public Health..

[15] Guan NC, Isa SM, Hashim AH, Pillai SK, Harbajan Singh MK (2015). Validity of the Malay Version of the Internet Addiction Test: a study on a group of medical students in Malaysia.. Asia Pac J Public Health..

[16] Musa R, Fadzil MA, Zain Z (2007). Translation, validation and psychometric properties of Bahasa Malaysia version of the Depression Anxiety and Stress Scales (DASS).. ASEAN J Psychiatr..

[17] Dang AK, Nathan N, Hoang Le QN (2018). Associations between internet addiction and physical activity among Vietnamese youths and adolescents.. Child Youth Serv Rev..

[18] Siste K, Hanafi E, Sen LT (2021). Implications of COVID-19 and lockdown on internet addiction among adolescents: data from a developing country.. Front Psychiatry.

[19] Ak J, Koruklu N, Yilmaz Y (2013). A study on Turkish adolescents internet use: possible predictors of internet addiction.. Cyberpsychol Behav Soc Netw..

[20] Fu SC, Pang NTP, Wider W (2022). Relationship between internet addiction, personality factors, and emotional distress among adolescents in Malaysia.. Children..

[21] Masaeli N, Farhadi H (2021). Prevalence of internet-based addictive behaviors during COVID-19 pandemic: a systematic review.. J Addict Dis..

[22] Zhao L, Li X, Yang Q (2023). The longitudinal association between internet addiction and depressive and anxiety symptoms among Chinese adolescents before and during the COVID-19 pandemic.. Front Public Health..

[23] Sangaran LN, Abdul Kadir A, Yaacob LH, Mohd Zin F, Azizah O (2022). Mental health and parental factors among adolescents during the COVID pandemic in Malaysia.. Int J Child Health Nutr..

[24] Shek DTL, Yu L (2016). Adolescent internet addiction in Hong Kong: prevalence, change, and correlates.. J Pediatr Adolesc Gynecol..

[25] Seyrek S, Cop E, Sinir H, Ugurlu M, Jenel S (2017). Factors associated with internet addiction: crosssectional study of Turkish adolescents.. Pediatr Int..

[26] Lee YS, Han DH, Kim SM, Renshaw PF (2013). Substance abuse precedes internet addiction.. Addict Behav..

[27] Lee J (2020). Mental health effects of school closures during COVID-19.. Lancet ChildAdolesc Health..

[28] Liu JJ, Bao Y, Huang X, Shi J, Lu L (2020). Mental health considerations for children quarantined because of COVID-19.. Lancet Child Adolesc Health..

[29] Zhai Y, Du X (2020). Mental health care for international Chinese students affected by the COVID-19 outbreak.. Lancet Psychiatry..

[30] Chang FC, Chiu CH, Lee CM, Chen PH, Miao NF (2014). Predictors of the initiation and persistence of internet addiction among adolescents in Taiwan.. Addict Behav..

